# Supplementary material for: Oral Dysbiosis Is Associated with the Pathogenesis of Aortic Valve Diseases
Source: Microorganisms. 2025 Jul 16;13(7):1677. doi: 10.3390/microorganisms13071677 (PMC12300341; doi:10.3390/microorganisms13071677)
Supplement: Supplementary file 1 [file microorganisms-13-01677-s001.zip › microorganisms-3713409-supplementary.pdf]

Supplementary Materials

Table S1. Detailed demographic data of the AS/AR patient.

						Transthoracic echocardiography findings					Valvular findings				
#No.	Age	Gender	Stage of Periodon-titis	Number of remaining teeth	Diagnosis	AVAi (cm²/m²)	LVEF	Peak PG	SV (ml)	Svi (ml/m²)	AR	MR	TR	PR	Structure
1	87	Female	IV	5	severe AS moderate	0.34	82	64	56	41	+	+	+	±	Calcification
2	77	Female	III	23	severe AS	0.62	74	95	90	58.4	-	-	±	-	Calcification
3	84	Female	NA	NA	moderate AS	0.64	64	22	39	25.9	±	++	++	±	Calcification
4	70	Female	IV	5	severe AS	0.26	65	88	49	29.7	±	±	—	—	Calcification Bicuspid valve
5	66	Male	IV	11	OMI moderate AS	0.41	40	42	39	26.5	—	+	+	+	Calcification
6	54	Female	III	21	severe AS	0.34	72	93	61	37.4	±	—	—	—	Calcification
7	66	Male	II	25	severe AS	ND	37	ND	141	94.1	+++	+	—	±	
8	65	Female	I	26	severe AS	0.4	44	54.17	47	32.6	+	±	+	±	Calcification
9	79	Female	NA	NA	severe AS	ND	45.2	56.3	ND	ND	+	+	±	—	Calcification
10	78	Female	NA	NA	severe AS	0.27	70	127.7	47	36.2	±	±	+	±	Calcification
11	58	Male	I	26	severe AS	0.36	34	94	78	44.5	+	±	±	++	Calcification Bicuspid valve s/o
12	74	Female	NA	NA	post PMI severe AS	0.32	63	116	52	36.6	+	+	±	±	Calcification
13	78	Female	NA	0	severe AS	0.55	69	53	55	44.6	+	+~++	+	+	Calcification
14	72	Female	NA	0	severe AS	0.4	59	71	51	33.1	+	++	+	±	Calcification
15	76	Male	II	24	moderate -se-vere AS mild PH	0.42	84	37	35	24	+	++	+	±	Calcification
16	79	Male	IV	12	severe AS	0.44	66	104	96	56	+~++	±	+	+	No abnormal findings
17	83	Male	III	23	severe AS	0.42	66	77	65	42.4	±	±	+	±	Calcification Bicuspid valve
18	85	Male	NA	0	moderate AS	0.86	59	57	112	69.1	++~+++	+	+	±	Calcification
19	79	Female	NA	NA	moderate-se-vere AR	ND	61	ND	106	82.6	++~+++	±	++	+	No abnormal findings
20	77	Male	IV	5	severe AS	0.48	34	73	93	53	+	+~++	±	+	Calcification
21	74	Male	IV	16	moderate-se-vere AR	ND	74	16.5	107	55.4	++~+++	-	-	+	Calcification
22	86	Female	IV	11	moderate-se-vere AS	0.58	67	56	57	42.8	+	+	+	±	Calcification
23	75	Male	I	23	severe AR	ND	60	ND	195	120	+++	+	±	+	Calcification
24	71	Male	NA	0	severe AS	0.4	24	79	73	39.9	++	+	+	-	Calcification Bicuspid valve
25	78	Female	IV	8	severe AS	0.53	67	54	91	53.2	+	+	+	±	Calcification
26	64	Male	III	22	severe AS	0.56	56	85	116	60.6	++	±	-	+	Bicuspid valve
27	76	Male	NA	0	severe AS	0.53	70	71	105	53	±	±	—	-	Calcification
28	84	Female	I	28	severe AS mild PH	0.38	65	102	79	59	±	+	±	±	Calcification
29	72	Male	I	27	severe AS post TAVI	ND	36	32	ND	ND	++	+	+	+	No abnormal findings
30	79	Female	IV	10	severe AS	0.28	62	129	56	38.4	—	+	+	—	No abnormal findings
31	65	Male	IV	11	severe AS	0.53	71	74	106	47	+	±	—	±	Calcification Bicuspid valve
32	61	Female	III	25	severe AS	0.38	75	83	71	40	-	-	±	-	Calcification Bicuspid valve

AS: aortic stenosis. AR: aortic regurgitation. AVA: aortic valve area. AVAi: AVA index. LVEF: left ventricular ejection fraction. Peak PG: peak pressure gradient. SV: stroke volume. SVi: stroke volume index. MR: mitral regurgitation. TR: tricuspid regurgitation. PR: pulmonary regurgitation. PH: pulmonary hypertension. OMI: old myocardial infarction. PMI: pacemaker implantation. TAVI: transcatheter aortic valve implantation. NA: not applicable. ND: not determined. In valvular

findings: -, no sign of regurgitation; ±, trace level of regurgitation; +, mild regurgitation; ++, moderate regurgitation; +++, severe regurgitation.

Table S2. Statistical analysis of Pielou's evenness index among dental plaque, tongue swab, and dissected aortic valve groups.

**All Groups**			
**Group**		**H**	**p-Value**
Plaque (n = 6)<br>Tongue (n = 6)<br>Valve (n = 6)		1.205	0.548
	**Pairwise**		
**Group 1**	**Group 2**	**H**	**p-Value**
Plaque (n = 6)	Tongue (n = 6)	0.923	0.337
	Valve (n = 6)	0.410	0.522
Tongue (n = 6)	Valve (n = 6)	0.641	0.423

Pielou's evenness index was compared among the three groups: dental plaque, tongue swab, and aortic valve samples (n = 6 each).

Table S3. Statistical analysis of Shannon's diversity index among dental plaque, tongue swab, and dissected aortic valve groups.

**All groups**			
**Group**		**H**	**p-Value**
Plaque (n = 6)<br>Tongue (n = 6)<br>Valve (n = 6)		0.889	0.641
	**Pairwise**		
**Group 1**	**Group 2**	**H**	**p-Value**
Plaque (n = 6)	Tongue (n = 6)	0.410	0.522
	Valve (n = 6)	0.000	1.000
Tongue (n = 6)	Valve (n = 6)	0.923	0.337

Shannon's diversity index was compared among dental plaque, tongue swab, and aortic valve samples (n = 6 each).

Table S4. PERMANOVA results based on unweighted UniFrac distances among dental plaque, tongue swab, and dissected aortic valve.

**Main Effect (Overall Comparison)**					
Source	Df	Sum of Squares	F	R^2	p
Site	2	1.2648	3.0248	0.2874	0.002
Residual	15	3.136		0.7126	
Total	17	4.4007		1	
Pairwise comparison with multiple testing correction					
Comparison	F	R^2	p	Adjusted p (Bonferroni)	
Plaque vs. Tongue	0.7600709	0.0706381	0.591	1	
Plaque vs. Valve	3.5467828	0.261874	0.003	0.009	
Tongue vs. Valve	3.6882548	0.2694467	0.002	0.006	

Beta diversity differences among dental plaque, tongue swab, and aortic valve samples (n = 6 each) were assessed using PERMANOVA based on unweighted UniFrac distance matrices. The overall comparison revealed a significant difference among the three groups (F = 3.0248, R^2 = 0.2874, p = 0.002). Pairwise comparisons with Bonferroni correction showed significant differences between plaque and valve samples (adjusted p = 0.009) and between tongue and valve samples (adjusted p

= 0.006). In contrast, no significant difference was observed between plaque and tongue samples (adjusted p = 1).

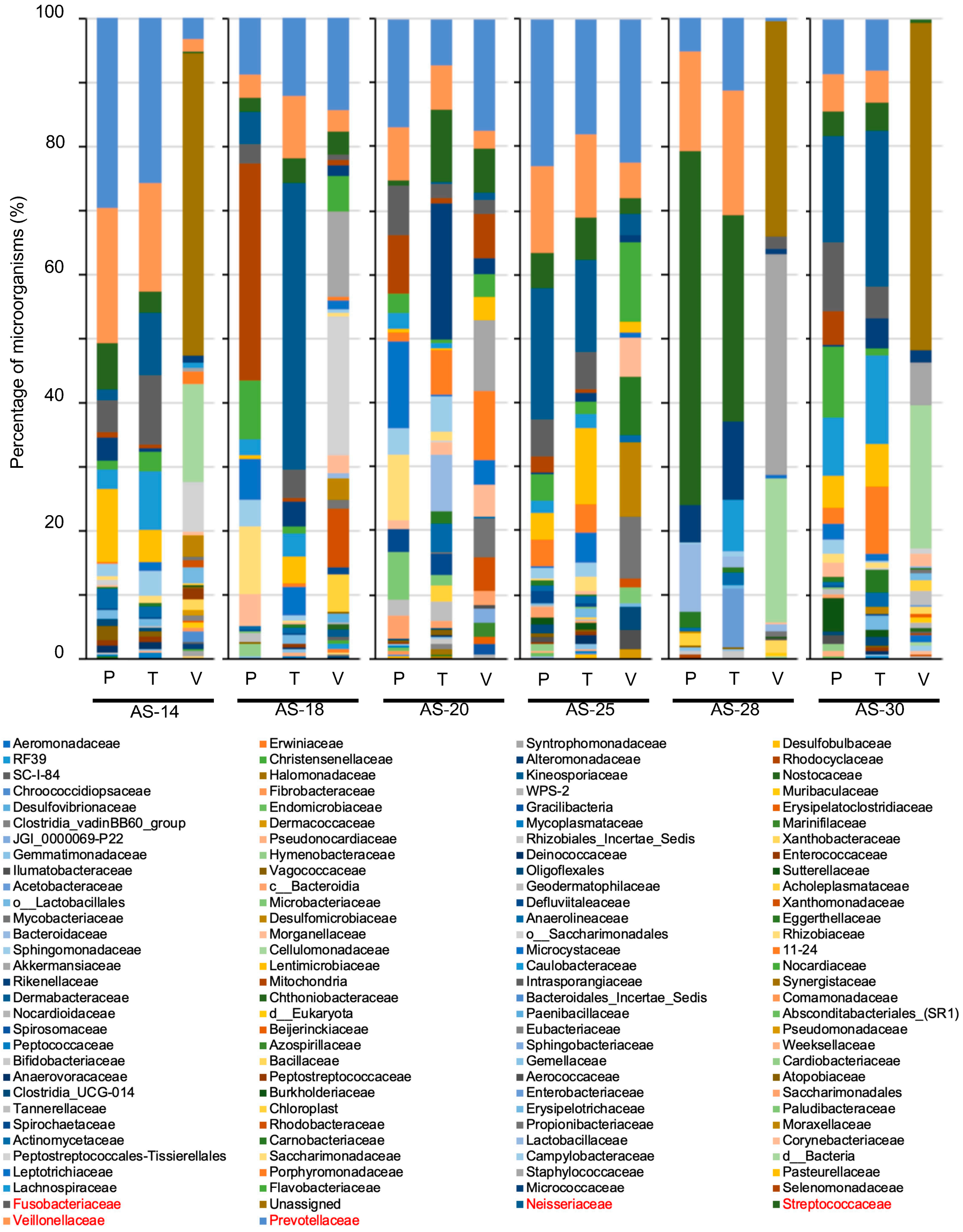

Figure S1. Composition of the bacterial microbiota at the genus level in samples of dissected aortic valve, dental plaque, and tongue swabs. P: dental plaque, T: tongue swab, V: the patient's dissected aortic valve. The proportion of unassigned samples was high in #14, 28, and 30. The PCR products were used as templates twice after the first PCR in these samples, which may have caused the artifacts. The most frequently detected bacteria were Prevotellaceae (■), Veillonellaceae (■), Streptococcaceae (■), Neisseriaceae (■), and Fusobacteriaceae (■), all common and abundant in the oral cavity (highlighted in red with an underscore).

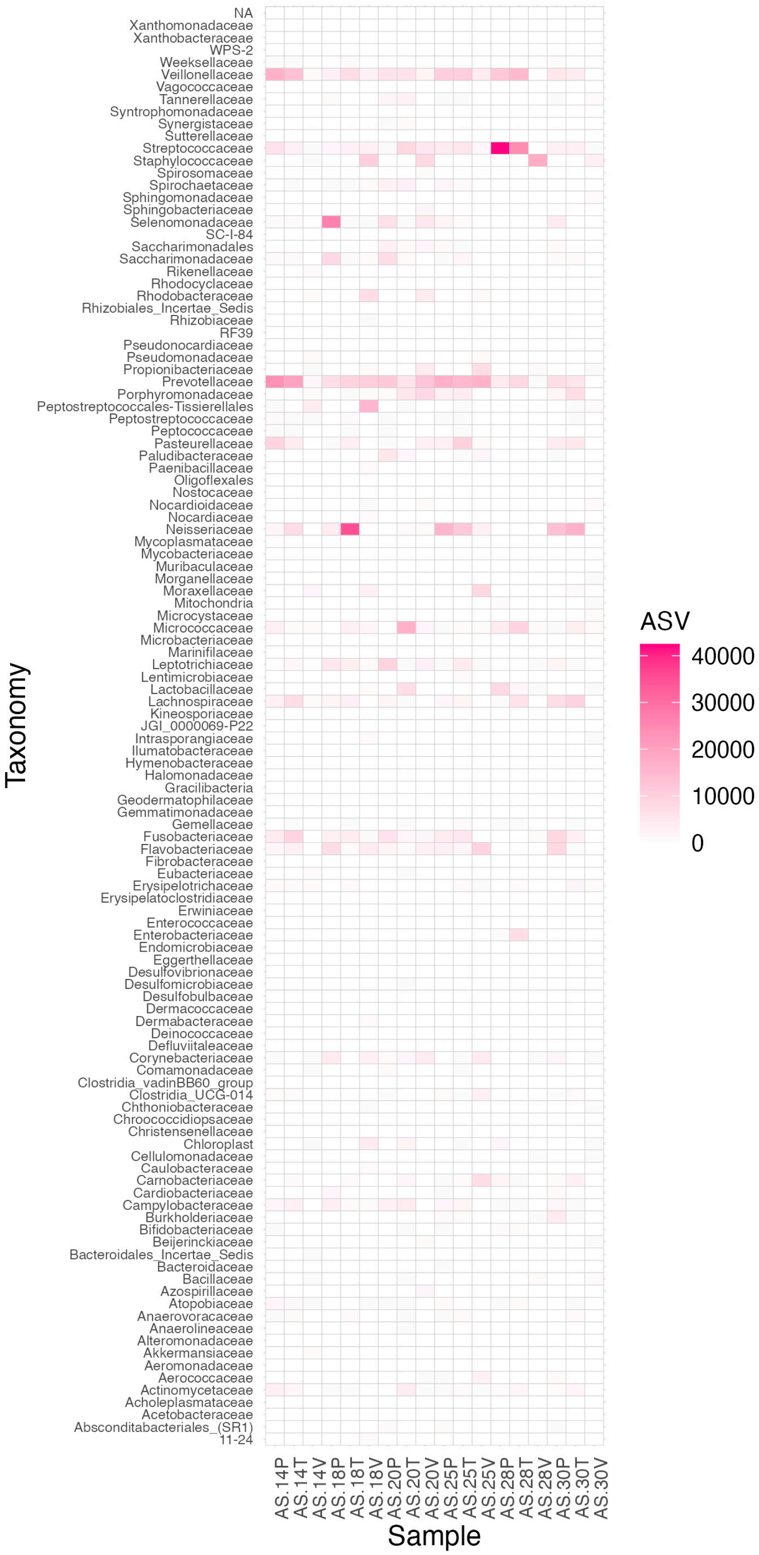

Figure S2. Heat map of the bacterial microbiota at the genus level in samples of dissected aortic valve, dental plaque, and tongue swabs. Bacterial composition of dental plaque, tongue swab, and dissected aortic valve was analyzed using 16S rRNA gene sequencing. Bacterial taxa identified at the genus level are shown on the vertical axis, while individual samples are displayed on the horizontal axis. The color gradient represents the feature counts of each genus in each sample, with darker shades indicating higher abundance.

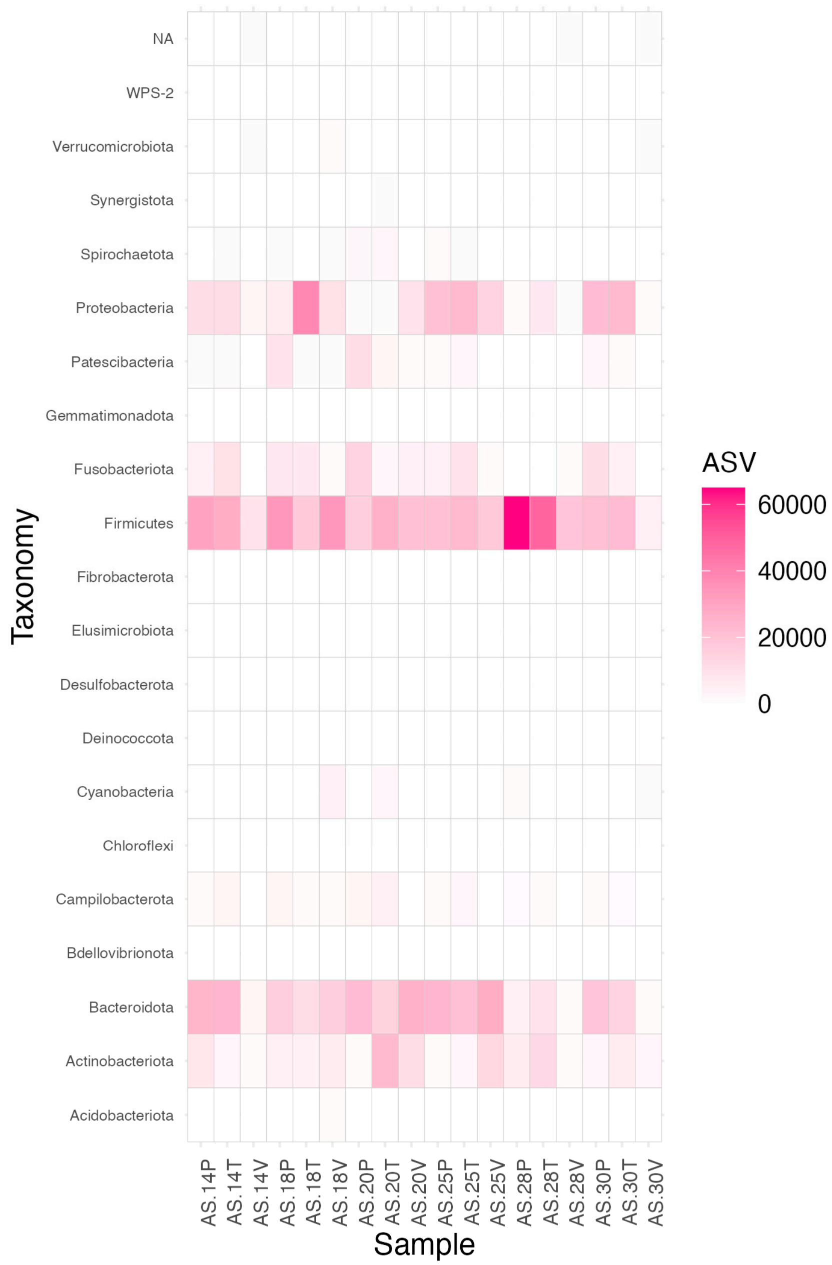

Figure S3. Heat map of the bacterial microbiota at the phylum level in samples of dissected aortic valve, dental plaque, and tongue swabs. Bacterial composition of dental plaque, tongue swab, and dissected aortic valve was analyzed using 16S rRNA gene sequencing. Bacterial taxa identified at the phylum level are shown on the vertical axis, while individual samples are displayed on the horizontal axis. The color gradient represents the feature counts of each genus in each sample, with darker shades indicating higher abundance.

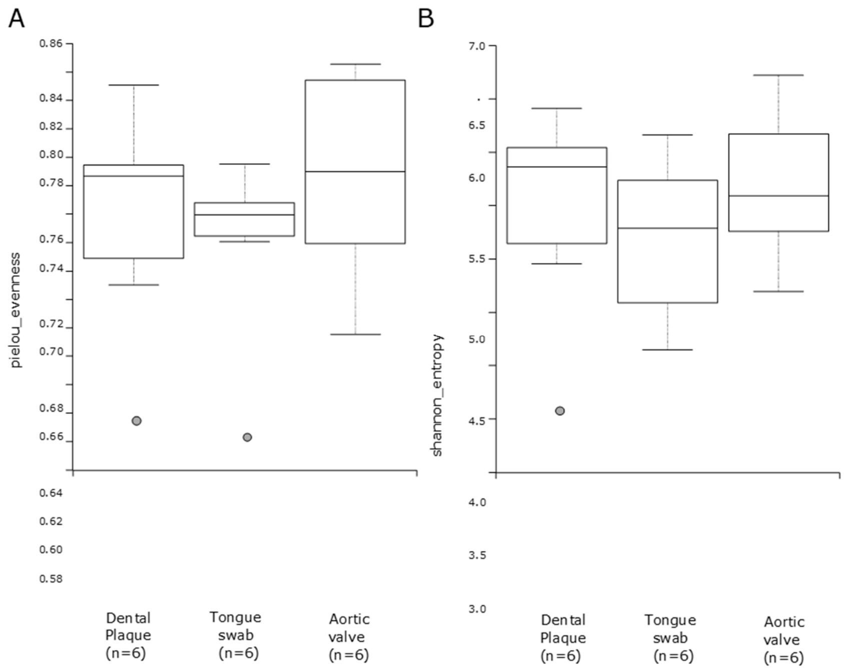

Figure S4. Comparison of alpha diversity for each sample group. (**A**) Peilou's evenness. (**B**) Shannon's entropy. The box plot represents the average values, and the dot represents the outlier. .

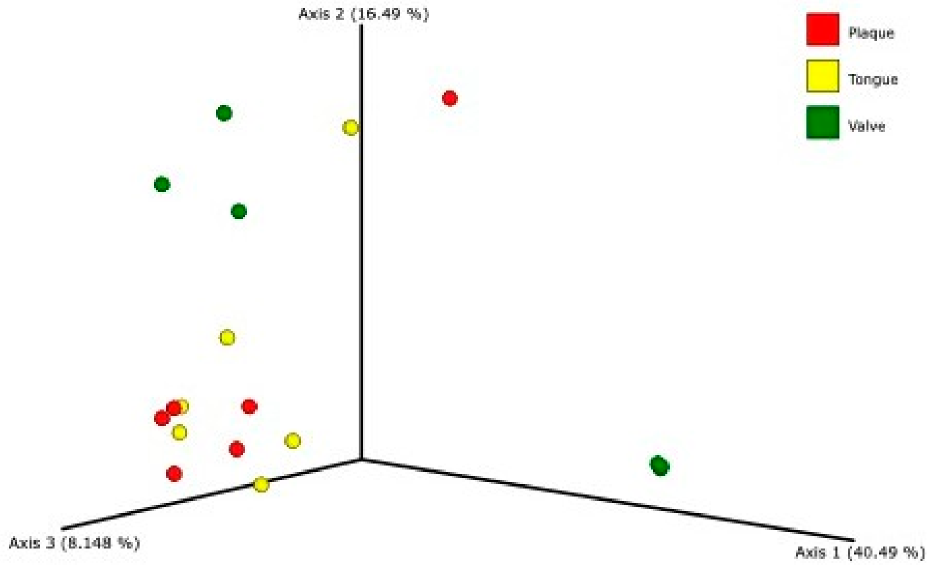

Figure S5. Principal coordinate analysis of each sample to examine beta diversity among groups. Red bubble: dental plaque, yellow bubble: tongue swab, green bubble: aortic valve. A significant difference in microbial composition was observed among the three groups (PERMANOVA: $F = 3.025$, $R^2 = 0.287$, $p = 0.002$), which indicates the difference of bacterial composition in each sample group.

A

	AS-14V		AS-18V		AS-20V	
Ranking	read count		read count		read count	
1	21743	Unassigned	9652	**g__Staphylococcus**	8038	**g__Staphylococcus**
2	7035	d__Bacteria	7813	**g__Anaerococcus**	5072	**g__Streptococcus**
3	2747	**g__Parvimonas**	6836	g__Finegoldia	4393	**Prevotella_oulorum**
4	1398	**Prevotella_sp.**	6272	g__Paracoccus	4385	g__Cutibacterium
5	1175	Solobacterium_moorei	4172	g__Chloroplast	4312	g__Centipeda
6	986	g__Acinetobacte	3231	**Prevotella_oulorum**	3801	g__Alloprevotella
7	807	**Porphyromonas_endodontalis**	3142	**Prevotella_denticola**	3623	**g__Porphyromonas**
8	724	Phocaeicola_abscessus	2467	g__Enhydrobacter	3042	g__Lawsonella
9	586	g__Enhydrobacter	2333	**g__Streptococcus**	2835	**Porphyromonas_pasteri**
10	525	g__Peptoniphilus	2276	**Capnocytophaga_ochracea**	2584	Haemophilus_parainfluenzae
11	497	f__Veillonellaceae	1608	**g__Veillonella**	2529	**Capnocytophaga_sputigena**
12	459	g__Pseudoramibacter	1529	**Capnocytophaga_gingivalis**	2053	__Paracoccus
13	458	**g__Peptostreptococcus**	1518	**Prevotella_nigrescens**	1862	**Prevotella_denticola**
14	450	d__Eukaryota	1507	Corynebacterium_tuberculostearicum	1790	g__Amaricoccus
15	441	**Bacteroidales_oral**	1037	g__Paenibacillus	1660	Candidatus_Saccharibacteria
16	441	Akkermansia_muciniphila	987	**Treponema_medium**	1613	**g__Fusobacterium**
17	431	g__Paracoccus	832	**g__Prevotella**	1599	g__Pedobacter
18	311	**g__Staphylococcus**	830	g__Peptoniphilus	1570	g__Skermanella
19	307	Dialister_invisus	801	**g__Rothia**	1489	**g__Porphyromonas**
20	294	g__Anaerobacillus	785	g__Cutibacterium	1397	g__Leptotrichia
21	274	**g__Pseudomonas**	772	g__Dermabacter	1389	**Veillonella_sp.**
22	270	Porphyromonas_sp.	680	**Prevotella_shahii**	1381	Leptotrichia_buccalis
23	244	Filifactor_alocis	612	**g__Fusobacterium**	1304	**g__Micrococcus**
24	240	g__Kocuria	600	g__Chthoniobacter	1161	g__Spirosoma
25	236	g__Anaerococcus	569	**Prevotella_loescheii**	1042	g__Alloprevotella

	AS-25V		AS-28V		AS-30V	
Ranking	read count		read count		read count	
1	7624	**Capnocytophaga_sp.**	17720	**g__Staphylococcus**	21298	Unassigned
2	7018	g__Cutibacterium	17324	Unassigned	9395	d__Bacteria
3	6683	Dolosigranulum_pigrum	11532	d__Bacteria	2757	**g__Staphylococcus**
4	6570	g__Alloprevotella	747	g__Anaerobacillus	966	Parabacteroides_goldsteinii
5	4680	g__Enhydrobacter	587	**Fusobacterium_periodonticum**	604	g__Lawsonella
6	4299	g__Lawsonella	549	Lactobacillus_iners	502	Kocuria_marina
7	3965	**Veillonella_sp.**	431	g__Cutibacterium	486	Cryptomeria_japonica
8	3895	**Prevotella_loescheii**	353	**g__Rothia**	450	Cyanothece_sp.
9	3867	Acinetobacter_baumannii	280	Bacillus_pseudofirmus	410	g__Faecalibaculum
10	3148	**Prevotella_nigrescens**	258	**g__Fusobacterium**	377	Sphingomonas_sp.
11	2875	**Prevotella_denticola**	256	d__Eukaryota	353	g__Nocardioides
12	2616	g__Clostridia_UCG-014	243	g__Streptobacillus	312	g__Anaerobacillus
13	2334	**g__Neisseria**	191	g__Alloprevotella	303	d__Eukaryota
14	2148	g__Abiotrophia	164	g__Lawsonella	275	**g__Streptococcus**
15	1828	**g__Streptococcus**	164	g__Cellulomonas	236	g__Cellulomonas
16	1633	g__F0058	162	g__Burkholderia-Caballeronia-Paraburkholderia	216	uncultured_Enterobacteriaceae
17	1448	**Capnocytophaga_ochracea**	121	f__Micrococcaceae	204	Cicer_arietinum
18	1182	f__Pasteurellaceae	103	**Fusobacterium_sp.**	194	g__Mitochondria
19	1121	g__Paracoccus	90	g__Neisseria	188	**g__Lactobacillus**
20	1053	g__Pseudomonas	58	**g__Porphyromonas**	186	uncultured_Spartobacteria
21	905	Kocuria_marina	49	g__Escherichia-Shigella	175	**g__Micrococcus**
22	697	g__Actinomyces	41	g__Aerosphaera	175	**g__Anaerococcus**
23	687	g__Streptobacillus	37	Murdochiella_asaccharolytica	171	g__Ezakiell
24	574	g__Solobacterium	32	**g__Streptococcus**	170	g__Methylobacterium-Methylorubrum
25	60	Corynebacterium_tuberculostearicum	31	g__Bradyrhizobium	158	Corynebacterium_tuberculostearicum

B

	AS-14P		AS-18P		AS-20P	
Ranking	read count		read count		read count	
1	11798	g__Veillonella	19179	Selenomonas_noxia	5688	g__Saccharimonadaceae
2	5142	Prevotella melaninogenica	6207	g__Saccharimonadaceae	5274	g__F0058
3	4948	g__Haemophilus	4906	Prevotella_shahii	4962	g__Fusobacterium
4	4441	g__Streptococcus	4340	Leptotrichia_hofstadii	4280	Anaeroglobus_geminatus
5	4345	Prevotella_nigrescens	3909	Corynebacterium_matruchotii	2607	Candidatus_Saccharibacteria
6	4042	Haemophilus_parainfluenzae	3777	Selenomonas_artemidis	2447	Leptotrichia_buccalis
7	3419	uncultured_Prevotella	3234	Capnocytophaga_granulosa	2336	g__Centipeda
8	3049	Fusobacterium_nucleatum	3005	Campylobacter_gracilis	2201	g__Leptotrichia
9	2588	g__Rothia	2275	g__Fusobacterium	2100	Leptotrichia_wadei
10	2141	g__Actinomyces	1958	g__TM7x;	1910	Campylobacter_gracilis
11	1704	Prevotella_histicola	1563	g__Streptococcus	1897	Prevotella_genomosp.
12	1700	Dialister_invisus	1526	Neisseria_bacilliformis	1797	Prevotella_nigrescens
13	1691	Prevotella_oris	1489	Capnocytophaga_ochracea	1582	Treponema_socranskii
14	1199	Prevotella_pallens	1392	Megasphaera_micronuciformis	1567	g__Tannerella
15	1195	Prevotella_genomosp.	1279	g__Veillonella	1486	Alloprevotella_tannerae
16	1128	Veillonella_parvula	1231	g__Selenomonas	1454	Prevotella_oralis
17	1107	Megasphaera_micronuciformis	1178	g__Capnocytophaga	1287	g__Prevotella
18	1079	g__Atopobium	1172	Tannerella_sp.	1259	g__TM7x
19	1070	Dialister_pneumosintes	1099	Selenomonas_sp.	1161	g__Leptotrichia
20	1040	Solobacterium_moorei	1097	g__Lachnoanaerobaculum	1052	g__Porphyromonas
21	1037	g__Alloprevotella	1082	g__Cardiobacterium	1050	Prevotella_marshii
22	890	Capnocytophaga_gingivalis	1036	Neisseria_elongata	997	Capnocytophaga_leadbetteri
23	864	g__Neisseria	901	g__Kingella	865	Corynebacterium_matruchotii
24	834	g__Fusobacterium	821	Prevotella_genomosp.	822	f__Selenomonadaceae
25	829	uncultured_Prevotella	629	Capnocytophaga_leadbetteri	728	Schwartzia_sp.

	AS-25P		AS-28P		AS-30P	
Ranking	read count		read count		read count	
1	9415	g__Neisseria	28191	Streptococcus_salivarius	9859	g__Neisseria
2	8062	g__Veillonella	14266	g__Streptococcus	4906	Fusobacterium_periodonticum
3	4745	Prevotella_melaninogenica	11266	Veillonella_atypica	4438	Prevotella_loescheii
4	4153	g__Fusobacterium	7423	Lactobacillus_fermentum	4225	g__Veillonella
5	3093	Streptococcus_sanguinis	3666	Prevotella_histicola	4170	g__Lautropia
6	2940	Porphyromonas_gingivalis	2925	g__Rothia	3702	Capnocytophaga_gingivalis
7	2929	Neisseria_elongata	1998	g__Granulicatella	3452	g__Fusobacterium
8	2901	g__Haemophilus	1340	Rothia_mucilaginosa	3404	g__Lachnoanaerobaculum
9	2498	Prevotella_loescheii	1263	g__Chloroplast	2793	g__Haemophilus
10	2269	Neisseria_oralis	688	g__Veillonella	2764	Neisseria_elongata
11	2047	Prevotella_nigrescens	480	Alloscardovia_omnicolens	1980	g__Selenomonas
12	1426	Prevotella_oris	464	g__Lactobacillus	1915	Streptococcus_sanguinis
13	1353	Prevotella_denticola	412	g__Mitochondria	1866	Capnocytophaga_ochracea
14	1287	Prevotella_intermedia	373	Prevotella_salivae	1560	Corynebacterium_matruchotii
15	1152	Candidatus_Saccharibacteria	287	g__Gemella	1458	g__Johnsonella
16	1140	Anaeroglobus_geminatus	279	Schaalia_odontolytica	1260	f__Selenomonadaceae
17	1037	Capnocytophaga_sp.	235	Campylobacter_concisus	1223	g__Campylobacter
18	1037	g__Clostridia_UCG-014	166	Oryza_sativa	1221	Prevotella_melaninogenica
19	987	Alloprevotella_tannerae	146	g__Atopobium	1193	Capnocytophaga_sputigena
20	832	f__Neisseriaceae	107	g__Actinomyces	1189	Capnocytophaga_leadbetteri
21	795	Capnocytophaga_leadbetteri	99	g__Klebsiella	1185	Porphyromonas_pasteri
22	791	g__Lautropia	96	Lactobacillus_gasseri	1175	g__Abiotrophia
23	750	Prevotella_genomosp.	83	g__Stomatobaculum	1098	g__Leptotrichia
24	714	Selenomonas_sputigena	83	g__Acinetobacter	1061	f__Lachnospiraceae
25	700	f__Lachnospiraceae;	80	Pyropia_yezoensis	1039	g__Johnsonella

C

	AS-14T		AS-18T		AS-20T	
Ranking	read count		read count		read count	
1	11585	**Prevotella_melaninogenica**	35218	**g__Neisseria**	15885	**g__Rothia**
2	8842	**g__Veillonella**	6809	**g__Veillonella**	5290	**g__Streptococcus**
3	8581	**Fusobacterium_periodonticum**	3194	g__Haemophilus	3976	**g__Campylobacter**
4	7567	**g__Neisseria**	3157	**Prevotella_melaninogenica**	3468	**Porphyromonas_gingivalis**
5	3733	**g__Lachnoanaerobaculum**	3080	**Fusobacterium_periodonticum**	2278	**g__Actinomyces**
6	3281	g__Haemophilus	2708	**g__Streptococcus**	2227	**Lactobacillus_reuteri**
7	3262	**Veillonella_atypica**	2405	**Prevotella_pallens**	2163	**Tannerella_forsythia**
8	3228	**Prevotella_pallens**	2139	g__Leptotrichia	2021	**Streptococcus_salivarius**
9	2640	**Campylobacter_concisus**	1880	**Prevotella_shahii**	1993	g__Chloroplast
10	2354	**g__Streptococcus**	1656	**Rothia_mucilaginosa**	1850	**Porphyromonas_endodontalis**
11	2295	**Capnocytophaga_gingivalis**	1489	g__Alloprevotella	1820	Lactobacillus_gasseri
12	2106	g__Oribacterium	1364	**g__Rothia**	1595	**Veillonella_atypica**
13	1895	**uncultured_Bacteroidetes**	1180	g__Oribacterium	1566	**g__Veillonella**
14	1175	Megasphaera_micronuciformis	1105	Leptotrichia_sp.	1470	Lactobacillus_fermentum
15	1034	Solobacterium_moorei	838	g__Solobacterium	1429	**Lactobacillus_salivarius**
16	956	Eubacterium_sulci	775	**Veillonella_atypica**	1423	g__Granulicatella
17	856	;g__Leptotrichia	715	g__Lachnoanaerobaculum	1200	g__F0058
18	792	**Prevotella_histicola**	631	**Campylobacter_concisus**	1133	**Treponema_lecithinolyticum**
19	772	**Schaalia_odontolytica**	597	**Capnocytophaga_gingivalis**	1116	Anaeroglobus_geminatus
20	711	;g__Catonella	538	g__Bergeyella	1089	**Streptococcus_anginosus**
21	667	**Prevotella_scopos**	529	**Schaalia_odontolytica**	1031	**g__Fusobacterium**
22	663	g__Atopobium	494	**Porphyromonas_pasteri**	1007	Corynebacterium_matruchotii
23	646	Haemophilus_parainfluenzae	450	**Streptococcus_salivarius**	959	**Schaalia_odontolytica**
24	607	**Prevotella_nanceiensis**	420	Selenomonas_sp.	927	Candidatus_Saccharibacteria
25	590	g__Peptococcus	417	**g__Fusobacterium**	809	**Prevotella_denticola**

	AS-25T		AS-28T		AS-30T	
Ranking	read count		read count		read count	
1	9703	**g__Neisseria**	13351	**g__Streptococcus**	13773	**g__Neisseria**
2	8093	**g__Veillonella**	10942	**Streptococcus_salivarius**	3550	g__Lachnoanaerobaculum
3	7379	**Haemophilus_parainfluenzae**	9978	**Veillonella_atypica**	3510	**Porphyromonas_pasteri**
4	4931	**Prevotella_pallens**	8167	**Rothia_mucilaginosa**	3265	**g__Rothia**
5	4911	**g__Streptococcus**	7005	**Prevotella_histicola**	3119	**Fusobacterium_periodonticum**
6	2450	**g__Fusobacterium**	6617	**g__Klebsiella**	3111	g__Streptococcus
7	2446	**Fusobacterium_periodonticum**	4582	g__Oribacterium	3098	Haemophilus_parainfluenzae
8	2324	**Prevotella_melaninogenica**	3304	Megasphaera_micronuciformis	2869	**Neisseria_elongata**
9	2292	g__Leptotrichia	1603	**g__Veillonella**	2754	g__Alloprevotella
10	1886	**Prevotella_jejuni**	1497	**Prevotella_salivae**	2699	**g__Porphyromonas**
11	1435	g__TM7x	1250	g__Stomatobaculum	2671	Lachnoanaerobaculum_umeaense
12	1302	**Neisseria_elongata**	1106	Lactobacillus_fermentum	2585	**g__Veillonella**
13	1240	g__Haemophilus	1051	**g__Rothia**	2464	g__Granulicatella
14	1207	**Porphyromonas_gingivalis**	749	Alloscardovia_omnicolens	1562	g__Solobacterium
15	1163	Solobacterium_moorei	703	**Schaalia_odontolytica**	1442	g__Haemophilus
16	1150	**Porphyromonas_pasteri**	682	**g__Actinomyces**	1335	g__Stomatobaculum
17	1125	g__Leptotrichia	635	g__Granulicatella	1297	**Schaalia_odontolytica**
18	1116	**g__Porphyromonas**	567	**Campylobacter_concisus**	1087	g__Clostridia_UCG-014
19	1042	g__Alloprevotella	431	Solobacterium_moorei	1011	**g__Porphyromonas**
20	1025	**g__Aggregatibacter**	351	g__Atopobium	929	g__Oribacterium
21	957	Megasphaera_micronuciformis	246	g__Lachnoanaerobaculum	767	g__Moraxella
22	916	Eubacterium_sulci	206	f__Enterobacteriaceae	764	**Veillonella_rogosae**
23	864	**g__Campylobacter**	196	g__Lactobacillus	742	**Prevotella_aurantiaca**
24	828	**Prevotella_intermedia**	139	g__Gemella	662	g__Johnsonella
25	819	**Prevotella_salivae**	81	**Actinomyces_graevenitzii**	623	g__Lautropia

Figure S6. Top 25 bacteria listed by read count in each sample. Bacteria related to the oral cavity are written in bold. The color of the background of the bacteria name was the same color as the bacteria, matching Sokransky's color code in Figure 2. (**A**) List of aortic valve samples. (**B**) List of dental plaque samples. (**C**) List of tongue samples.
